# Trifluridine/tipiracil regimen in combination with bevacizumab for metastatic colorectal cancer in the third line: an expert opinion

**DOI:** 10.3389/fonc.2024.1502185

**Published:** 2025-01-22

**Authors:** Carmine Pinto, Sara Lonardi, Evaristo Maiello, Erika Martinelli, Michele Prisciandaro, Lisa Salvatore, Andrea Sartore-Bianchi, Mario Scartozzi, Giuseppe Aprile, Chiara Cremolini, Alberto Sobrero

**Affiliations:** ^1^ Oncologia Medica, Comprehensive Cancer Centre, AUSL-IRCCS di Reggio Emilia, Reggio Emilia, Italy; ^2^ Department of Oncology, Veneto Institute of Oncology IOV-IRCCS, Padova, Italy; ^3^ Oncology Unit, Foundation IRCCS Casa Sollievo della Sofferenza, San Giovanni Rotondo, FG, Italy; ^4^ Oncologia Medica, Dipartimento di Medicina di Precisione, Università degli Studi della Campania “L. Vanvitelli “, Napoli, Italy; ^5^ Medical Oncology Department, Fondazione IRCCS Istituto Nazionale dei Tumori, Milano, Italy; ^6^ Medical Oncology, Università Cattolica del Sacro Cuore, Rome, Italy; ^7^ Medical Oncology, Comprehensive Cancer Center, Fondazione Policlinico Universitario Agostino Gemelli, IRCCS, Rome, Italy; ^8^ Division of Clinical Research and Innovation, ASST Grande Ospedale Metropolitano Niguarda, Milano, Italy; ^9^ Department of Oncology and Hemato-Oncology, University of Milano, Milano, Italy; ^10^ Oncologia Medica, Azienda Ospedaliero Universitaria e Università degli Studi di Cagliari, Cagliari, Italy; ^11^ Department of Medical Oncology, AULSS8 Berica, Vicenza, Italy; ^12^ Department of Translational Research and New Technologies in Medicine and Surgery, University of Pisa, Pisa, Italy; ^13^ Oncologia Medica, IRCCS Ospedale Policlinico San Martino, Genova, Italy

**Keywords:** trifluridine/tipiracil, bevacizumab, third line therapy, metastatic colorectal cancer, efficacy, safety

## Abstract

The prolongation of survival along with the preservation of quality of life, possibly avoiding harmful cumulative toxicities, is the primary therapeutic aim for patients with metastatic colorectal cancer (mCRC) in the third-line setting. Several therapeutic options are now available, although some differences across countries in drug approval and the optimal therapeutic sequencing associated with each peculiar patient subgroup represent a clinical challenge for oncologists. Among various options, the SUNLIGHT trial showed how the combination of trifluridine/tipiracil (FTD/TPI) with bevacizumab is effective with an easily manageable toxicity profile compared to FTD/TPI alone. Of note, the efficacy is confirmed independently from KRAS mutational status and also for patients who had breaks in anti-vascular endothelial growth factor (anti-VEGF) therapy. Herein, we describe the current state of the art in the landscape of treatments after the second progression in mCRC. Based on a critical review of the literature aimed to guide clinicians in their daily decision-making, we point out that the combination of FTD/TPI with bevacizumab produces a clinical benefit in unselected mCRC patients. Therefore, the FTD/TPI plus bevacizumab regimen can represent a new standard of care for the treatment of patients with refractory mCRC who have progressed after two lines of therapy.

## 1 Introduction

Colorectal cancer (CRC) is recognized as the third most common solid tumor worldwide with an incidence of 1.1 million cases/year. Furthermore, CRC is the second leading cause of tumor death (1).

In Europe (updated statistics in 2018), CRC has the second-highest events of tumor death (2).

Within all CRC cases, approximately 15%–30% present already with metastases, and 20%–50% of patients will develop metastases after an initial localized disease (3).

Systemic treatment of metastatic colorectal cancer (mCRC) has improved considerably over the past 20 years. First- and second-line combinations of 5-fluorouracil (5-FU), oxaliplatin, and irinotecan, with or without anti-angiogenic and/or anti-epidermal growth factor receptor (anti-EGFR) antibodies, were approved shortly after the turn of the millennium. After a pause of approximately 10 years, further progress has been made in the treatment of mCRC.

In the last years, treatment outcomes, in terms of both efficacy and quality of life (QoL), have improved impressively (4). Among possible factors of this improvement, especially for mCRC, is the rise of available therapies and their efficacy (5). Beyond anti-EGFR therapies in RAS/BRAF wild-type tumors, other targeted therapies have indeed emerged, including immune checkpoint inhibitors for deficient mismatch repair (dMMR) tumors, BRAF inhibition for BRAFV600E mutant, HER2 blockade in HER2-positive, and anti-KRAS G12C inhibitors in KRAS G12C mutant cancers (6PMID: 35472088). In fact, there is a growing trend in patients who undergo a third line worldwide (3, 6, 7). Considering these premises, optimizing the choice and the right sequencing of treatments after the second line for mCRC is a medical need. The latest National Comprehensive Cancer Network (NCCN), European Society for Medical Oncology (ESMO), and American Society of Clinical Oncology (ASCO) guidelines (3, 6, 7) stressed this issue, i.e., that sequencing criteria have to consider the type of patient and his/her previous medical history to improve therapy outcomes while preserving safety and QoL and avoiding also the potential impact of harmful cumulative toxicities from previous treatments.

## 2 Preclinical rationale for the FTD/TPI and bevacizumab combination

The anti-angiogenic drug bevacizumab interacts with the vascular endothelial growth factor (VEGF) and therefore on the tumor vasculature, favoring the effectiveness of chemotherapy through the “normalization” of blood flow (8). These biological data were then confirmed in randomized clinical trials, which reported how the introduction of bevacizumab, in combination with fluoropyrimidine-based chemotherapy regimens, improves the efficacy of the same regimens in the treatment of mCRC. This combination now represents a standard of care (SoC) in both first- and second-line therapies (9–12). Trifluridine/tipiracil (FTD/TPI) is a combination of a) the thymidine-based nucleoside analog (FTD), which has been reported to act through inhibition of thymidylate synthase and incorporation into the DNA, ultimately leading to DNA damage and cell death; and (b) the TPase inhibitor (TPI), which prevents the rapid degradation of FTD, thus extending its short half-life. As a result, FTD/TPI remains in the body longer and can therefore be administered at a lower dose. The new generation fluoropyrimidine, FTD/TPI, has shown its efficacy as monotherapy in patients with mCRC in the third line and beyond, obtaining a clinical benefit even in patients previously treated with fluoropyrimidines (13). This result can be attributed to the different mechanism of action of FTD/TPI compared to other fluoropyrimidines. In fact, trifluridine, in addition to inhibiting the proliferation of tumor cells by blocking the enzyme thymidylate synthase, carries out its main function through direct incorporation into the DNA of dividing tumor cells, causing damage at the DNA level and consequently death. Furthermore, the addition of tipiracil prevents the metabolism of trifluridine, leading to an increase in its bioavailability (13). These results led to the approval of monotherapy for adult mCRC patients who have already relapsed after two previous lines such as fluoropyrimidine (5-FU/capecitabine)-, oxaliplatin-, and irinotecan-based chemotherapy and anti-VEGF therapy and for RAS wild-type mCRC patients treated with anti-EGFR therapy (13). The toxicity profile and pharmacokinetics, characterized by metabolism and excretion pathways that do not interfere with those of many other neoplastic drugs, make FTD/TPI a candidate drug for the development of new combination therapies (14, 15). Therefore, the combination with an anti-angiogenic drug such as bevacizumab, capable of improving the efficacy of a fluoropyrimidine-based chemotherapy regimen, constituted a solid rationale for the development of this regimen in the third-line setting of mCRC. The pharmacological rationale of the combination of FTD/TPI and bevacizumab was confirmed in preclinical models, and in particular on mice bearing SW48 (KRAS wild type) and HCT116 (KRAS mutated) human colorectal carcinoma xenografts. FTD/TPI and bevacizumab administered individually confirmed an inhibition of tumor growth, but the combined treatment showed synergistic anti-tumor activity, consequently superior to the results obtained with the individual drugs. The levels of phosphorylated trifluridine present in the tumor, in fact, were found to be higher with the addition of bevacizumab, confirming how the latter is responsible for the increase in the bioavailability of FTD/TPI and, consequently, for the ability to inhibit the growth of the tumor volume. This effect was demonstrated in both models, therefore being independent of the mutation of the RAS13 gene (16).

## 3 Clinical studies supporting the FTD/TPI ( ± bevacizumab combination) in advanced mCRC in third line

FTD/TPI in the phase III RECOURSE study, which compared this drug with a placebo, produced a median overall survival (OS) of 7.2 months versus 5.2 months [hazard ratio (HR) 0.69, 95% confidence interval (95% CI) 0.59–0.81], a median progression-free survival (PFS) of 2.0 months versus 1.7 months (HR 0.48, 95% CI 0.41–0.57), an overall response rate (ORR) of 1.6% versus 0.4%, and a disease control rate (DCR) of 44% versus 16% (17, 18). Similar results occurred in the TERRA study with comparable survival (mOS and mPFS of 7.8 and 2.0 months, respectively, and a DCR of 44.1%) (19).

A systematic review and a meta-analysis of real-life studies concluded that when physicians choose to administer FTD/TPI monotherapy as salvage-line treatment in refractory mCRC patients, the efficacy from RECOURSE may be reproduced (20).

The first phase 1–2 clinical trial evaluating the combination of FTD/TPI and bevacizumab in 25 Asian patients with mCRC refractory or intolerant to standard therapies (fluoropyrimidines, irinotecan, oxaliplatin, anti-VEGF therapy, and anti-EGFR therapy for tumors with wild-type KRAS) was the C-TASK FORCE trial (21). In this study, the recommended dose of the combination for the phase 2 study was defined, which was FTD/TPI 35 mg/m^2^ administered orally twice daily on days 1–5 and 8–12 and bevacizumab 5 mg/kg administered as a 30-minute intravenous infusion every 2 weeks. The combination demonstrated a good tolerability profile and promising anti-tumor activity in the first randomized phase 2 study, developed in four Danish centers, in 93 patients with chemorefractory mCRC. In this study, a statistically significant improvement of 2 months in PFS was highlighted (mPFS 4.6 months vs. 2.6 months; HR 0.45, *p* = 0.0015), and OS had a median of 9.4 months (21).

Based on these results, the international phase III SUNLIGHT study (NCT04737187) was designed to evaluate the efficacy of the FTD/TPI regimen in combination with bevacizumab compared with FTD/TPI alone in patients with mCRC treated with two previous lines of therapy containing fluoropyrimidines, irinotecan, oxaliplatin, and anti-VEGF and/or anti-EGFR antibody therapy in patients with wild-type RAS tumors (22). The population included in the study, in the period from 2020 to 2022, was mainly Caucasian, of which 64% was European, and they had been pretreated with two previous regimens. As part of these regimens, 100% of patients received fluoropyrimidines, 100% irinotecan, 98% oxaliplatin, and 72.4% an anti-VEGF drug. RAS mutations were found in 70% of patients, and 43% of cases had a time from diagnosis of metastatic disease to randomization of less than 18 months, elements that characterize a population with a worse prognosis (23).

The combination of FTD/TPI and bevacizumab allowed a statistically and clinically significant improvement in OS of 3.3 months compared to the SoC, with an absolute median value of 10.8 months compared to 7.5 with FTD/TPI alone, with a reduction in 39% risk of death (HR 0.61, 95% CI 0.49–0.77, *p* < 0.001), and 1-year survival was 43% and 30%, respectively. These results are close to the survival medians obtained in second-line studies with a survival advantage in favor of the combination regardless of the site of the primary tumor and previous exposure to bevacizumab (24–26).

Of note, these results are confirmed also for patients who had breaks in anti-VEGF therapy (27). In particular, this *post-hoc* analysis of the SUNLIGHT trial showed that patients who never received anti-VEGF had mOS of 7.4 months for the FTD/TPI alone and 16.4 months for the combination; in patients who had received anti-VEGF only in the first line, mOS was 7.2 (monotherapy) and 10.4 (combination). For patients who received the anti-VEGF therapy in the second line only, mOS was 8.2 months (monotherapy) and 9.0 months (combination), and in patients who received anti-VEGF in both the first and second lines, mOS was 6.9 months (monotherapy) and 9.4 months (combination) (27).

In addition, in all phase II and III studies of the first and second lines with antiangiogenic agents plus chemotherapy doublets, all antiangiogenics have always produced greater efficacy in RAS wt tumors compared to RASm ones. In this regard, a recent meta-analysis concluded that treatment with FTD/TPI leads to a benefit in terms of survival—compared to placebo—regardless of KRAS codon 12 or 13 mutations in previously treated mCRC (28), confirming the evidence from preclinical studies and phase 2 clinical studies; the OS advantage was observed regardless of the RAS mutational status. Furthermore, a *post-hoc* analysis of the SUNLIGHT trial stressed further that KRAS mutational status had no detrimental effect on OS in this setting for patients who received FTD/TPI as a single agent and in combination with bevacizumab (29). Patients treated with FTD/TPI in combination with bevacizumab showed prolonged OS compared to patients who received FTD/TPI as a single agent, regardless of KRAS mutational status. Of note, in the subgroup with KRAS mutant, mOS was 9.4 months and 11.3 months for patients without KRAS mutation for the combined regimen. In patients treated with FTD/TPI as a single agent, mOS was 7.2 and 7.1 (29).

To elucidate, we conducted a review of clinical studies on FTD/TPI monotherapy and FTD/TPI in combination with bevacizumab to compare and discuss the efficacy results of these two treatment options (**Tables 1**, **2**).

**Table 1:** Clinical trials reporting on FTD/TPI monotherapy.

Survival/endpoints	Yoshino et al., 2012 (30)	Mayer et al., 2015 (17)	Xu et al., 2018 (19)	Takahashi et al., 2018 (31)	Bachet et al., 2020 (32)	Pfeiffer et al., 2020 (21)	Prager et al., 2023 (23)
n*	112	534 (533)	271 (261)	30	793	47	246
Setting	Pretreated ≥2 CHT lines	Pretreated ≥2 CHT lines	Pretreated ≥2 CHT lines	Refractory ≥65 years	Pretreated ≥2 CHT lines	Refractory or intolerant to CHT	Pretreated ≤2 CHT lines
Study phase and design	Phase 2 double-blind, randomized, placebo-controlled	Phase 3 double-blind study, TAS-102 or placebo (2:1 ratio)	Phase 3 randomized, double-blind, placebo-controlled	Phase 2 single-arm, open-label	Phase 3b open-label, single-arm	Phase 2 open-label, randomized	Phase 3 randomized (1:1 ratio) FTD/TPI plus bevacizumab or FTD/TPI alone
PFS at 3 months	NA	NA	NA	NA	45%	NA	NA
PFS at 6 months	NA	NA	NA	NA	18%	NA	16%
PFS median (months)	2.0 (95% CI 1.9–2.8)	2.0 (95% CI 1.9–2.1)	2.0 (95% CI 1.9–2.8)	2.3 (95% CI 1.9–4.3)	2.8 (95% CI 2.7–2.9)	2.6 (95% CI 1.6–3.5)	2.4 months (95% CI 2.1–3.2)
OS at 6 months	NA	27%**	NA	NA	NA	NA	61%
OS median (months)	9.0 (95% CI 7.3–11.3)	7.1 (95% CI 6.5–7.8)	7.8 (95% CI 7.1–8.8)	5.7 (95% CI 3.7–8.9)	NA	6.7 (95% CI 4.9–7.6)	7.5 months (95% CI 6.3–8.6
ORR	1%	1.6%	1.1%	0%	2.3%	NA	1.2%
DCR	43% (54%)	44%	44.1% (on 261 patients)	57%	34.4%	51%	NA
Follow-up median (months)	11.3 (IQR 10.7–14.0)	11.8 (NA)	13.8 (95% CI 13.1–15.3)	NA	NA	10 (IQR 6.8–14.0)	13.6 months (IQR 12.7 to 15.9)

CHT, chemotherapy; CI, confidence interval; DCR, disease control rate; FTD/TPI, trifluridine/tipiracil; IQR, interquartile range; NA, not available; ORR, overall response rate; OS, overall survival; PFS, progression-free survival.

*Treated with FTD/TPI.

**At 12 months.

**Table 2:** Clinical trials reporting on FTD/TPI in combination with bevacizumab.

Survival/endpoints	Kuboki et al., 2017 (33)	Yoshida et al., 2021 (34)	Satake et al., 2020 (35)	Takahashi et al., 2021 (36)	Pfeiffer et al., 2020 (21)	Prager et al., 2023 (23)
n*	25	31	44	97	46	246
**Setting**	Pretreated ≥1 CHT lines	Pretreated ≥2 CHT lines	Refractory or intolerant to CHT	Refractory or intolerant to CHT	Refractory or intolerant to CHT	Pretreated ≥2 CHT lines
**Study phase and design**	Phase 1–2 open-label, single-arm	Phase 2 open-label, single-arm	Phase 1b–2 open-label, single-arm	Phase 2 open-label, single-arm	Phase 2 open-label, randomized	Phase 3 randomized (1:1 ratio) FTD/TPI plus bevacizumab or FTD/TPI alone
**PFS at 3 months**	60% (95% CI 39–79)**	NA	40.9% (95% CI 26.3%–56.8%)**	NA	NA	NA
**PFS at 6 months**	NA	NA	NA	NA	NA	43%
**PFS median (months)**	3.7 (95% CI 2.0–5.4)	4.5 (95% CI 1.8–7.1),	4.29	3.7 months (95% CI 2.6–4.1)	4.6 (95% CI 3.5–6.5)	5.6 (95% CI 4.5–5.9)
**OS at 6 months**	NA	NA	NA	NA	NA	77%
**OS median (months)**	11.4 (95% CI 7.6–13.9)	9.2 (95% CI 5.5–12.8)	10.86	9.1 (95% CI 7.4–10.5),	9.4 (95% CI 7.6–10.7)	10.8 (95% CI 9.4 –11.8)
**ORR**	0%	6.5%	NA	3.1%	NA	6.1%
**DCR**	64%	65.6%	59.1%	60.8%	67%	NA
**Follow-up median (months)**	11.4 (IQR 7.4–15.6)	NA	NA	15.8	10 (IQR 6.8–14.0)	14.2 (IQR 12.6–16.4)

CHT, chemotherapy; CI, confidence interval; DCR, disease control rate; FTD/TPI, trifluridine/tipiracil; IQR, interquartile range; NA, not available; ORR, overall response rate; OS, overall survival; PFS, progression-free survival.

*FTD/TPI in combination with bevacizumab

**At 12 months.

In clinical trials, for cohorts treated with FTD/TPI alone, mPFS and mOS were 2.0–2.8 and 6.7–9.0 months, respectively. DCR ranged from 34.4% to 57%. Referring to studies for mCRC patients treated with the combination of FTD/TPI and bevacizumab, the overall improvement in mPFS and mOS were 3.7–4.6 and 2.8 and 9.1–11.4, respectively. DCR was from 59.1% to 67%.

Regarding safety, FTD/TPI plus bevacizumab has a similar safety profile to that of FTD/TPI monotherapy, except for a higher rate of grade ≥ 3 neutropenia. However, the increased occurrence of this adverse event may not be clinically significant, as absolute discontinuation rates due to toxicities were low with both the combination therapy and monotherapy, indicating that both regimens were generally well tolerated. Of note, the risk of treatment discontinuation due to toxicity was similar despite a longer treatment duration with the combination therapy than the one with monotherapy (median, 4.9 months versus 2.4 months) (37). To date, the relative increase in granulocyte colony-stimulating factor (G-CSF) use in the study compared to clinical practice may be related both to the use that was permitted in the trial and to the greater attention of the researchers to the control of neutropenia that usually occurs in a clinical study.

In the SUNLIGHT trial, risks were similar between the two cohorts for grade ≥ 3 febrile neutropenia, asthenia/fatigue, diarrhea, nausea, and vomiting (21).

Recently, the RAMTAS/IKF643 phase III study compared FTD/TPI plus ramucirumab with FTD/TPI alone in 428 patients with chemotherapy-refractory mCRC. The mOS (primary endpoint) was 7.46 months for patients treated with ramucirumab compared with 7.06 months for patients on SoC only (HR 0.871, 95% CI 0.708–1.073, *p* = 0.1941). Improvements in OS were observed in female patients (HR 0.71, 95% CI 0.52–0.98, *p* = 0.04) and those with left-sided tumors (HR 0.77, 95% CI 0.60–1.070, *p* = 0.05). Median PFS was significantly prolonged with ramucirumab compared with standard of care alone (2.37 months versus 2.07 months; HR 0.774, 95% CI 0.636–0.949, *p* = 0.011), and there was also a significant improvement in DCR (39.4% versus 31.6%, *p* = 0.0336). This study did not meet its primary endpoint. These results are not surprising considering that the patients were heavily pretreated, with 62.2% undergoing >2 prior lines of therapy (38). These results also confirm that the optimal positioning of the FTD/TPI plus bevacizumab regimen is in the third line of therapy.

## 4 Expert opinion

The best practice for mCRC in the third line is usually based on national/international guidelines and impacted by possible differences across countries in drug approval. However, there are universally valid criteria that should be taken into account for the best treatment choice, such as patient and disease characteristics and expected toxicity (37). For disease-specific characteristics, physicians consider molecular biomarkers and previous treatment received, and for patient-specific characteristics, the main items are performance status and global medical history (39–41).

To date, the number of available options for patients who relapse after two lines of therapy is increasing. However, each of these options has limitations in its application. One choice can be treatment with monoclonal antibody (mAb) cetuximab or panitumumab if the patient has not received these drugs previously (42). This option is seldom adopted, as anti-EGFR mAbs are commonly used in earlier lines. The same concept can be applied to immunotherapy for mCRC patients with dMMR/MSI-H or POLE/PLOD1 mutation, for which we have very strong evidence of efficacy in the first and second lines for this small patient subset (42–44).

Patients who progress while on treatment with anti-EGFR-based therapy can be resistant to further anti-EGFR treatment, but evidence suggests that the anti-EGFR-resistant clones decay, thereby opening the potential for rechallenge or reintroduction in later lines of treatment, especially if the treatment choice is guided by analysis of ctDNA (45, 46). However, the possibility of adopting this option in clinical practice is still limited by several issues: even though mentioned in clinical guidelines (3), no phase 3 randomized trials have been reported. In addition, the application of liquid biopsy in clinical practice, an essential tool to select the right candidates for this strategy, has not been fully implemented yet. Another choice is biomarker-targeting agents as the ones against BRAFV600E, HER2, KRASG12C, and NTRK (6, 47). However, most patients with mCRC do not present other actionable genetic alterations. Therefore, for the “all comers”, the approved available treatments include regorafenib and fruquintinib or FTD/TPI as a single agent or in combination with bevacizumab (6, 42).

Regorafenib is one of the current oral therapies recommended in the third-line treatment of mCRC. Regorafenib is an oral tyrosine kinase inhibitor targeting angiogenesis, the tumor microenvironment, and tumor immunity (47), and it is approved for the treatment of mCRC after progression on standard therapies (48), based on results of the phase III CORRECT trial and CONCUR trial (49, 50).

Fruquintinib, a tyrosine kinase inhibitor, has been shown to be effective in heavily pretreated mCRC progressing after FTD/TPI, regorafenib, or both (51). Preclinical studies have shown that fruquintinib inhibits with high selectivity VEGFR 1-2-3, leading to a blockade in the angiogenesis process, but also acts, with weak inhibition, on RET, FGFR-1, and c-kit kinases. Fruquintinib demonstrated good efficacy and tolerance in chemorefractory mCRC in two phase III trials: FRESCO and FRESCO-2 (52, 53). These results led to the Food and Drug Administration (FDA) approval of fruquintinib for pretreated mCRC patients who received prior fluoropyrimidine-, oxaliplatin-, and irinotecan-based chemotherapy. To date, in the FRESCO-2 study, patients were all pretreated with FTD/TPI and/or regorafenib (53), and, as a consequence, the European Medicines Agency (EMA) indication is different from FDA approval: fruquintinib is not actually a third-line option in Europe.

FTD/TPI, as monotherapy or in combination with bevacizumab, is also considered a SoC in the third-line setting of mCRC. In the SUNLIGHT phase 3 clinical trial, a clinically meaningful gain in OS in favor of this regimen has been shown compared to FTD/TPI alone. The advantage in terms of OS matches the preservation of QoL. As stated before, these results are very remarkable considering that prolonging OS while maintaining a good QoL is the main therapeutic goal in this setting.

In addition, the results of the SUNLIGHT study are in line with real-life experiences published, including evidence also in fragile patients due to age and/or comorbidities (30).

The main international guidelines recommend the use of the FTD/TPI plus bevacizumab combination in clinical practice. The last ESMO guidelines attribute a level of IA evidence to the combination of FTD/TPI and bevacizumab, with an ESMO-MCBS v1.1 scale score of 4, higher than that of all the third-line options reported in the different subgroups (with the exception of encorafenib–cetuximab for patients with BRAF V600E mutation, which received the same score). For a non-curative setting, a score of 4 out of 5 is a harbinger and indicative of substantial clinical benefit (3).

Taking into account these data, some considerations can be made.

First, the combination of fluoropyrimidines and antiangiogenics, in particular bevacizumab, represents a cornerstone for the treatment of mCRC.

The combination of agents from these two classes has always been the basis of first- and second-line regimens, and thanks to the SUNLIGHT study, it is confirmed that this regimen also produces important and favorable outcomes for patients in the third-line setting. Antiangiogenics monotherapy in advanced lines has not demonstrated effective improvements. FTD/TPI alone has been demonstrated to induce an improvement in survival outcomes in mCRC patients undergoing third line and beyond due to a different mechanism of action from the fluoropyrimidines that allows to overcome resistance when the drug is used in first and second approaches. However, the improvements obtained with FTD/TPI as a single agent appear less impressive when compared with those reached using the combination regimen.

Second, maintaining an inhibition of angiogenesis over time is very important in mCRC. The addition of bevacizumab to FTD/TPI improved the OS of the treatment in the third line, preserving QoL without a clinically relevant increase of toxicity. In the SUNLIGHT trial, the combination regimen increased the risk of severe neutropenia, mainly due to the longer duration of treatment. Nevertheless, only three patients receiving FTD/TPI plus bevacizumab developed febrile neutropenia (FN). To date, FN is easily managed by dose delay (median 7 days), and overall, no treatment-related deaths have occurred (21).

In addition, a *post-hoc* subgroup analysis of SUNLIGHT has shown that the benefit of adding bevacizumab to FTD/TPI is independent regardless of the previous use of bevacizumab in other lines, and it has been confirmed also in those patients who have received bevacizumab as part of their last previous regimen or in both previous first- and second-line treatments (27).

The importance of maintaining the angiogenesis inhibition of the patients’ therapeutic pathway is now more relevant than ever considering the other therapeutic options currently available, namely, regorafenib and fruquintinib. Therefore, in a hypothetical therapeutic algorithm, the chance to offer patients regorafenib and fruquintinib after FTD/TPI + bevacizumab can ensure angiogenic inhibition maintenance over time. Finally, in the era of precision oncology, the FTD/TPI plus bevacizumab regimen can be positioned in the mCRC treatment algorithm for patients in whom there is an actionable clinical target considering the availability of drugs and the approval status in each country (54, 55).

## 5 Conclusions

Selecting the most appropriate treatment in the third-line mCRC setting poses several challenges. Therefore, it is important for oncologists to understand and differentiate between available treatment options and to communicate the benefits and challenges of each of them to patients.

The pharmacological combination of FTD/TPI and bevacizumab showed synergistic anti-tumor activity first in preclinical models. This was then confirmed in subsequent clinical trials. Of note, outcomes reported in the third-line setting underscored a statistically and clinically significant improvement in OS, preserving QoL with manageable toxicity. The SUNLIGHT trial showed the clinical benefit of a combination regimen versus an active comparator agent in unselected mCRC patients. Therefore, the FTD/TPI plus bevacizumab regimen can represent a new effective SoC for the treatment of patients with refractory mCRC who have progressed after two lines of therapy.
